# An Unusual Presentation of Diverticular Colovesical Fistula: Acute Renal Failure With Bilateral Emphysematous Pyelonephritis

**DOI:** 10.7759/cureus.92456

**Published:** 2025-09-16

**Authors:** Kristen Williams, Mackenzie Owen, Andrea Honeycutt

**Affiliations:** 1 Internal Medicine, University of North Carolina School of Medicine, Chapel Hill, USA; 2 Internal Medicine, WakeMed Raleigh Hospital, Raleigh, USA

**Keywords:** acute renal failure and hemodialysis, asymptomatic colovesical fistula, bacterial pyelonephritis, colovesical fistula, colovesical fistula diverticulitis, diverticulitis, emphysematous pyelonephritis (epn), recurrent fevers, sigmoid diverticulitis

## Abstract

Colovesical fistula (CVF) is an abnormal connection between the urinary system and the bowel. It is a known complication of acute diverticulitis. Common symptoms include pneumaturia, fecaluria, and abdominal pain, amongst others. In this case, we report a rare case of acute renal failure due to a relatively asymptomatic colovesical fistula causing left-sided emphysematous pyelonephritis complicated by renal abscess. After appropriate surgical treatment and antibiotic therapy, the CVF, pyelonephritis, and abscess resolved. Unfortunately, renal function did not return to baseline, likely due to injury and poor functioning of the left kidney. Urology is considering radical nephrectomy to prevent recurrent of future infection. This case highlights a previously unknown and severe patient presentation of CVF and signifies the importance of maintaining a high index of suspicion of a fistula when a patient presents with recurrent urinary tract infections, acute renal failure, and colon pathology.

## Introduction

A colovesical fistula (CVF) is an abnormal connection between the urinary system and the bowel. Common causes of this include diverticulitis, IBD (Crohn's disease), and gynecologic and colorectal malignancy. Less common causes include iatrogenic etiology from colorectal or urinary surgery. Presenting signs and symptoms can include pneumaturia, fecaluria, abdominal pain, and bacteriuria on urinalysis [1-4]. Notably, CVF can be diagnosed clinically in patients with these symptoms even without imaging or laboratory confirmation. Nevertheless, imaging such as computed tomography and/or cystography with contrast can be utilized for diagnosis. Management can be conservative with antibiotics and steroids or can require surgical intervention, depending upon the extent of the disease and the status of the patient.

Here, we describe the case of a 49-year-old man who initially presented with oliguria that progressed to anuria. Abdominal and pelvic CT revealed a CVF. After sigmoid colectomy, bladder repair, and antibiotic treatment, he progressively improved; however, his kidney function has not normalized five months after presentation, and urology is considering radical nephrectomy for definitive management.

## Case presentation

A 49-year-old man was admitted to our hospital with bilateral flank pain, progressive oliguria leading to anuria, dyspnea, and fatigue for 1.5 weeks. His past medical history was significant for HTN and obesity. Prior to this, he was performing labor-intensive work for 8-9 hours for three days without proper hydration. He started experiencing fevers/chills that resolved with Tylenol. He also reported bilateral flank pain, headache, dark urine, and two days of non-bloody diarrhea. Three days leading up to the hospitalization, he became anuric, later accompanied by dyspnea, dry mouth, loss of appetite, and 15lb weight gain.

On admission, the patient was tachycardic with a heart rate of 113 bpm and afebrile. On his physical exam, he had dry mucous membranes and no costovertebral tenderness. Tables 1-3 show the patient's initial complete blood count, complete metabolic profile, and urinalysis. Notably, his initial testing revealed an elevated brain natriuretic peptide level of 765 pg/dL and normal creatine kinase.

**Table 1:** Initial complete blood count with differential on admission CBC: complete blood count; WBC: white blood cells; RBC: red blood cells; RDW: red cell distribution width

Initial CBC with Differential	Patient Values	Reference Ranges
WBC	14	3.6 - 11.2 K/ul
RBC	4.3	4.06 - 5.63 M/ul
Hemoglobin	13.9	12.5 - 16.3 g/dl
Hematocrit	40	37 - 47 %
Mean cell volume	92	74 - 96 fl
Mean cell hemoglobin	32	24 - 33 pg
Mean cell hemoglobin concentration	35	33 - 36 g/dl
RDW	15	12.3 - 17.0 %
Platelet count	164	150 - 450 K/ul
Neutrophils	80	43 - 77 %
Lymphocytes	12	16 - 44 %
Monocytes	8	5 - 13 %
Eosinophils	0	1 - 8 %
Basophils	0	0 - 1 %
Neutrophils absolute	11.2	1.8 - 7.8 K/ul
Lymphocytes absolute	1.7	1.0 - 3.0 K/ul
Monocytes absolute	1.1	0.3 - 1.0 K/ul
Eosinophils absolute	0	0.0 - 0.5 K/ul
Basophils absolute	0	0.0 - 0.1 K/ul
Nucleated RBCs	0	/100 WBC

**Table 2:** Initial complete metabolic panel on admission BUN: blood urea nitrogen; ALT: alanine aminotransferase; AST: aspartate aminotransferase

Complete Metabolic Panel	Patient Values	Reference Ranges
Sodium	124	136 - 145 mmol/L
Potassium	5.7	3.5 - 5.1 mmol/L
Chloride	84	98 - 107 mmol/L
CO2	22	21 - 31 mmol/L
BUN	112	7 - 25 mg/dL
Creatinine	10.54	0.70 - 1.30 mg/dL
Glucose, random	100	70 - 199 mg/dL
Calcium, total	8.8	8.6 - 10.3 mg/dL
Osmolality (calculated)	285	270 - 295 mOsm/kg
Anion gap	18	4 - 12
Albumin	2.9	3.5 - 5.7 g/dL
Bilirubin, total	1.6	0.3 - 1.0 mg/dL
Alkaline phosphatase	149	34 - 104 IU/L
ALT	51	7 - 52 IU/L
AST	47	13 - 39 IU/L
Protein, total	6.2	6.4 - 8.9 g/dL
Albumin/globulin ratio	0.9	1.2 - 2.3

**Table 3:** Initial Urinalysis and Urine Electrolytes RBC: red blood cells; WBC: white blood cells; HPF: high power field; CFU: colony-forming units

Urinalysis and Urine Electrolytes	Patient Values	Reference Ranges
Color	Bloody	Light yellow or yellow
Urine clarity	Turbid	Clear
Urine specific gravity	1.02	1.003 - 1.035
Urine pH	8	5.0 - 8.0
Urine albumin	3+	Negative
Urine glucose screen	1+	Negative
Urine ketones	Trace	Negative
Urine bilirubin	Moderate/Large	Negative
Urine hemoglobin	Large	Negative
Urine leukocyte esterase	Moderate	Negative
Urine nitrite	Positive	Negative
Urine urobilinogen	2	0.2 - 1.0 mg/dL
Urine bacteria	3+	Trace /HPF
Urine RBC	3 to 20	<3 /HPF
Urine WBC	>50	<5 /HPF
Urine squamous epithelial cells	<10	<10 /HPF
Urine WBC clumps	4+	None
Protein/creatinine ratio	38.46	<0.2 mg/mg creatinine
Urine sodium	108	mmol/L
Urine reflex culture	>100,000 CFU E.Coli	No reference range

**Figure 1:**
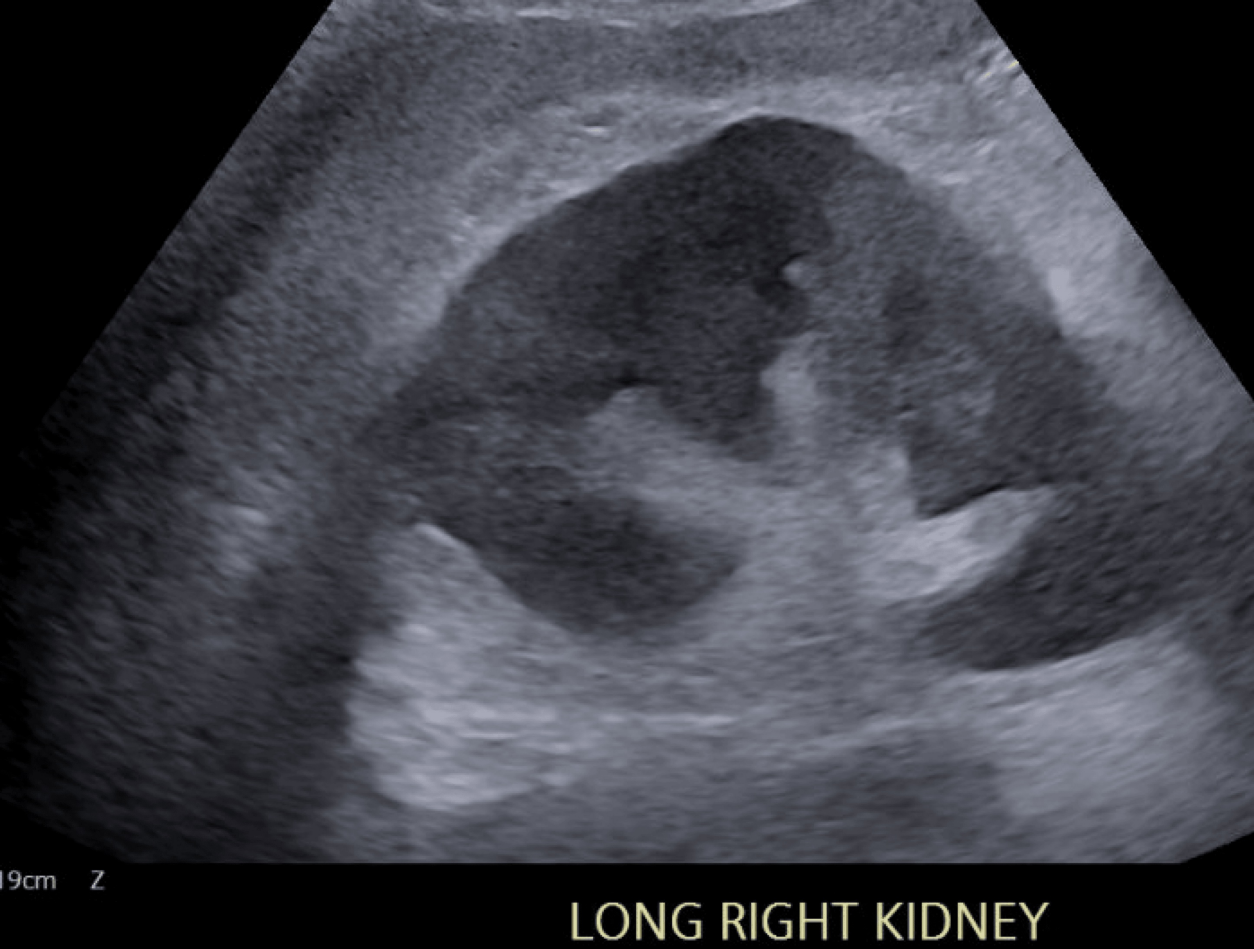
Longitudinal view of the right kidney on renal ultrasound The renal cortex appears homogeneous, the medullary pyramids are distinguishable, and the central echogenic renal sinus is well defined.

**Figure 2:**
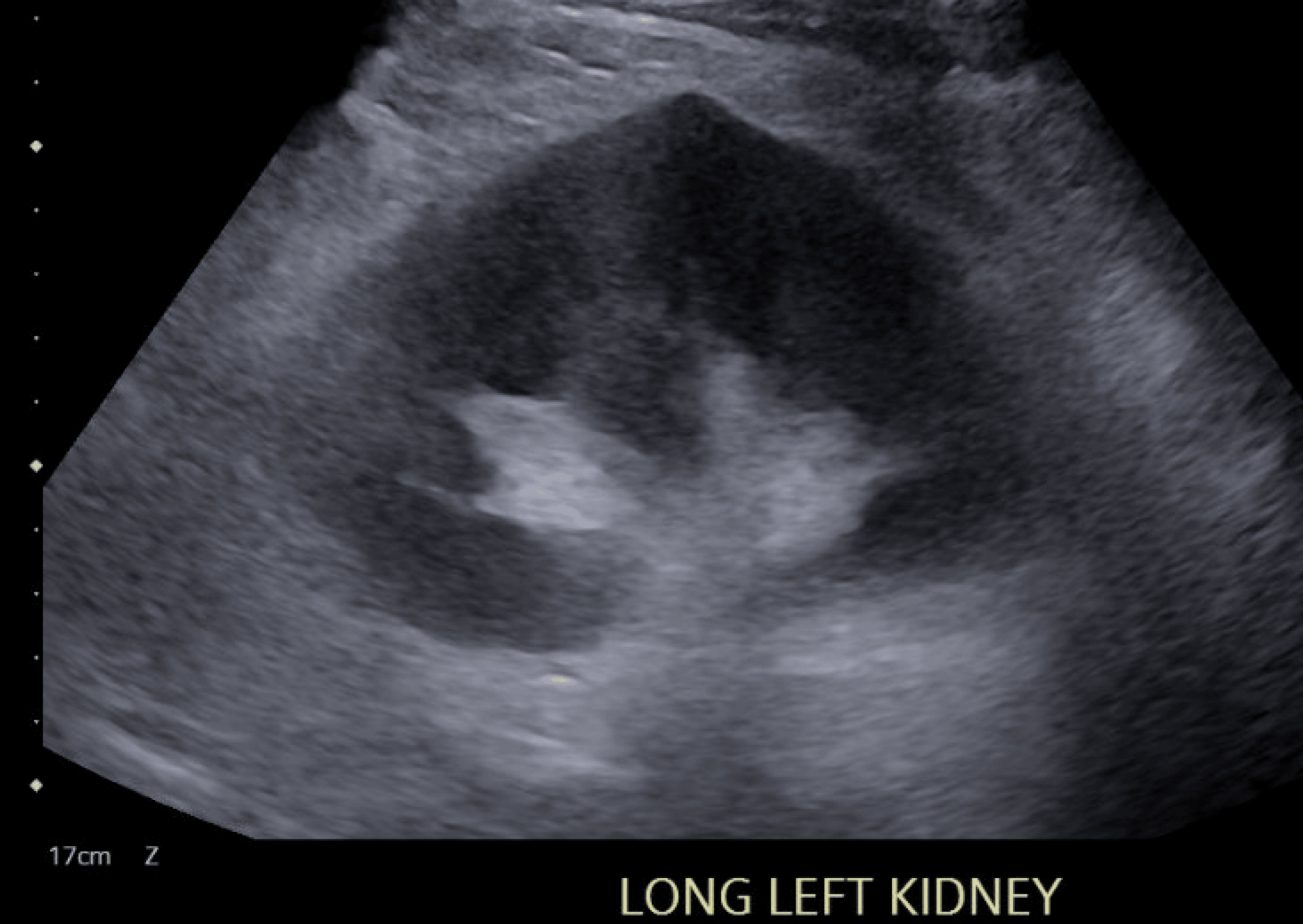
Longitudinal view of the right kidney on renal ultrasound The renal cortex appears homogeneous, the medullary pyramids are distinguishable, and the central echogenic renal sinus is well defined.

**Figure 3:**
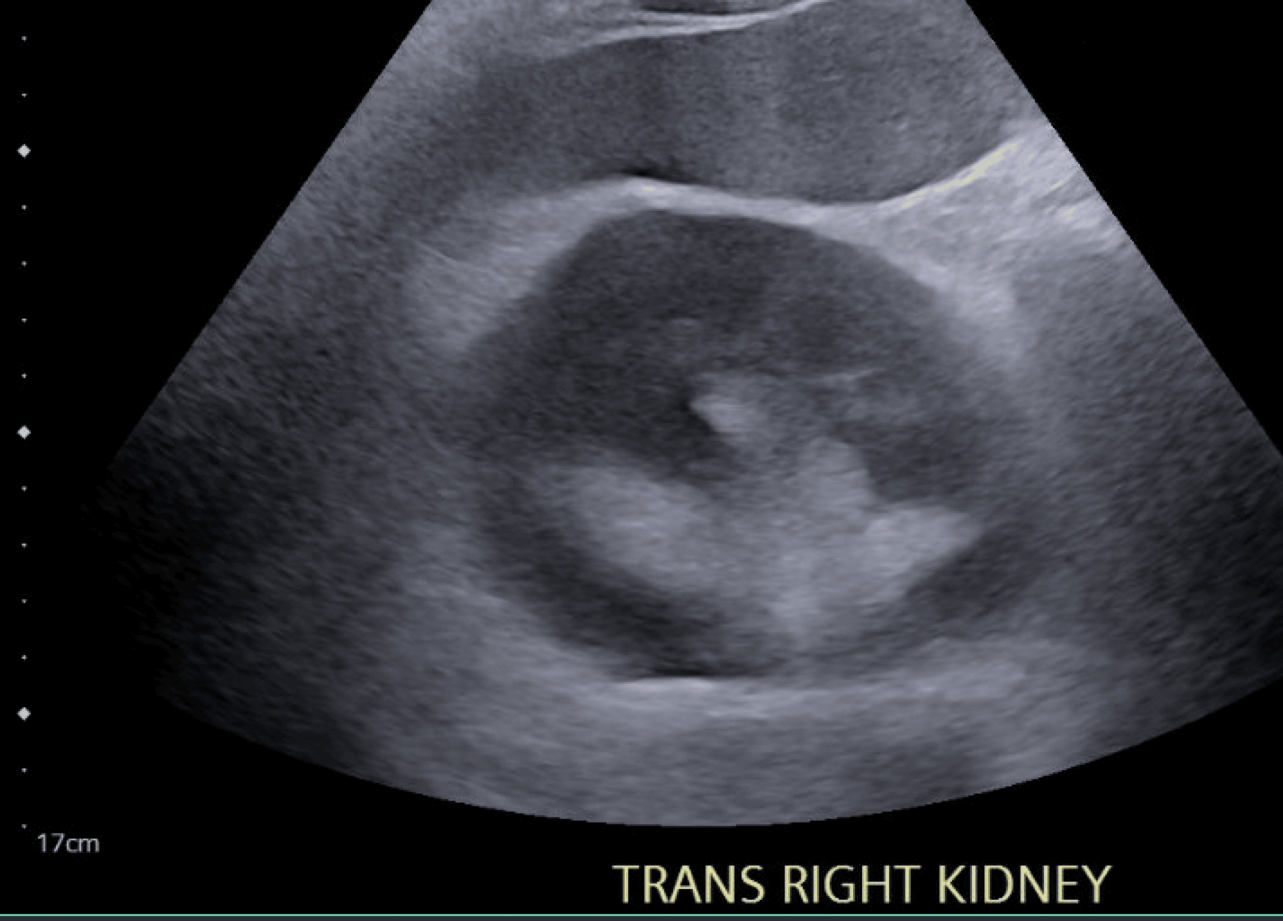
Transverse view of the right kidney which shows a normal echotexture and parenchymal thickness without hydronephrosis, abscess, or nephrolithiasis.

**Figure 4:**
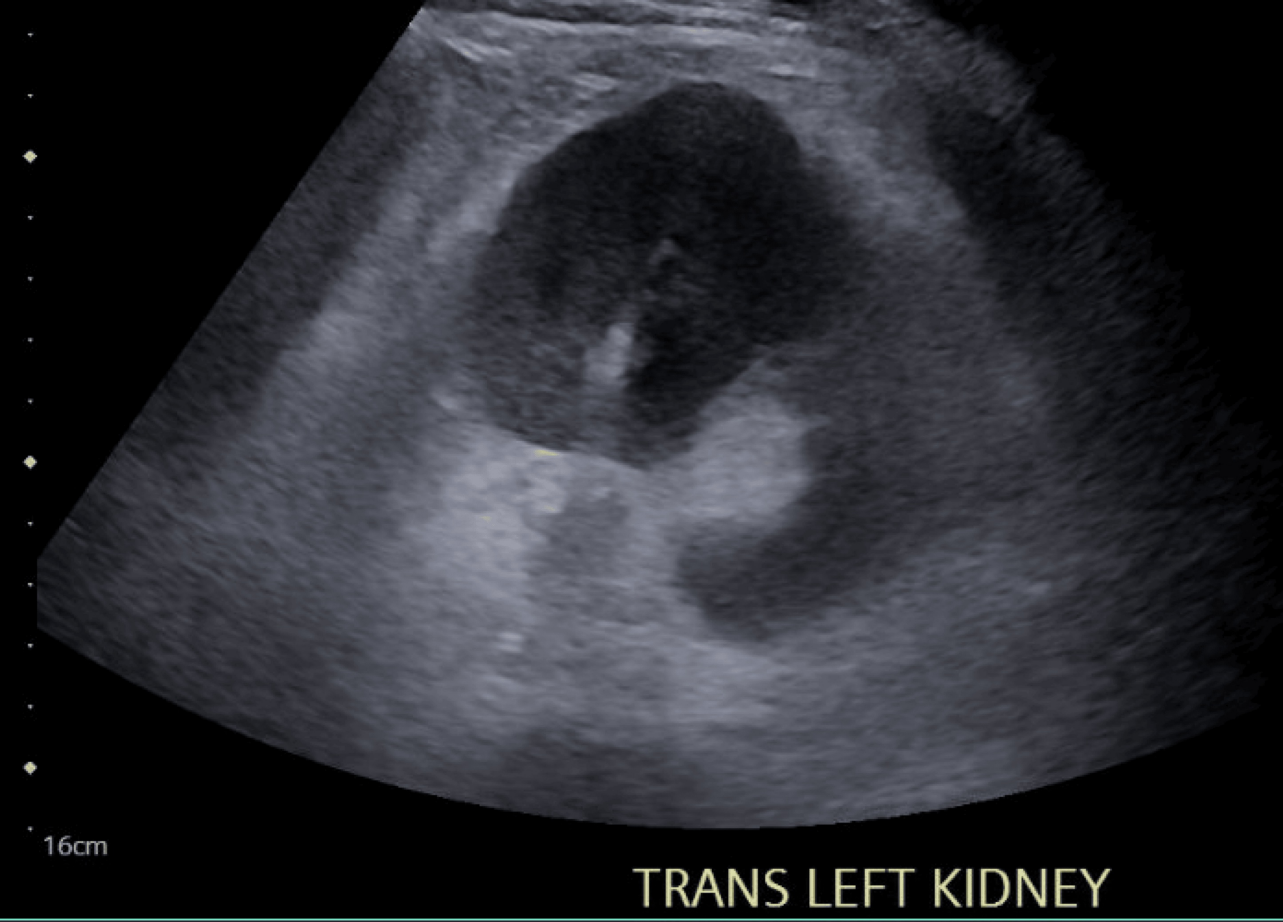
Transverse view of the left kidney which shows a normal echotexture and parenchymal thickness without hydronephrosis, abscess, or nephrolithiasis.

Due to the concern for severe acute renal failure, a renal ultrasound was completed. Figures 1-4 display the renal ultrasound that showed normal echotexture and parenchymal thickness without hydronephrosis, solid mass, or nephrolithiasis.

**Figure 5:**
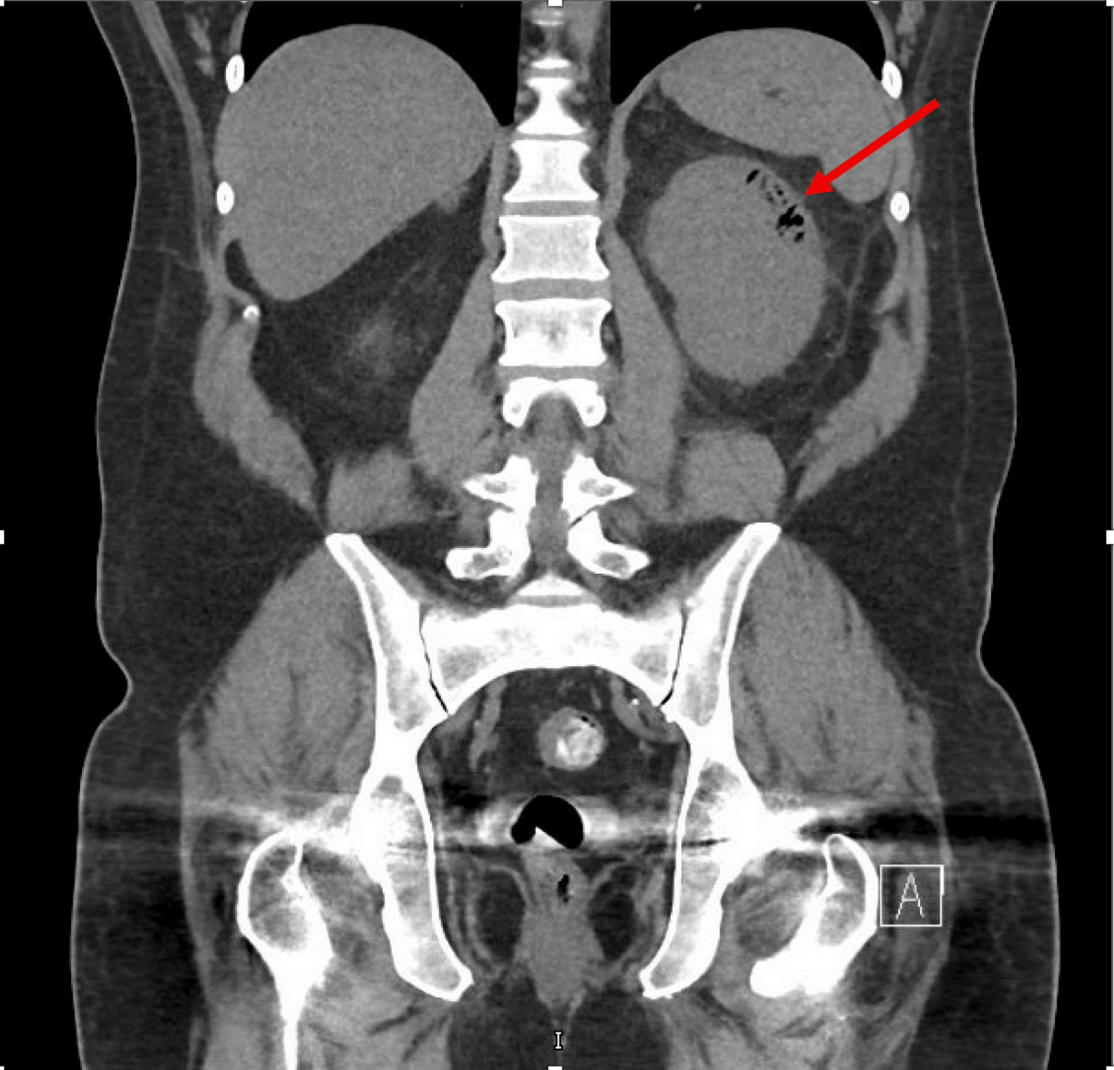
Left emphysematous pyelonephritis found on CT-guided biopsy

Given the risk of rapidly progressive glomerulonephritis, nephrology recommended a renal biopsy. The patient underwent a CT-guided biopsy, which demonstrated emphysematous changes involving the left kidney with perinephric stranding. Figure 5 shows an image from the CT scan for evaluation of kidney stones, which demonstrated a mildly enlarged right kidney with extensive areas of air throughout the left renal cortex, indicating emphysematous pyelonephritis along with thickening of the sigmoid colon.

**Figure 6:**
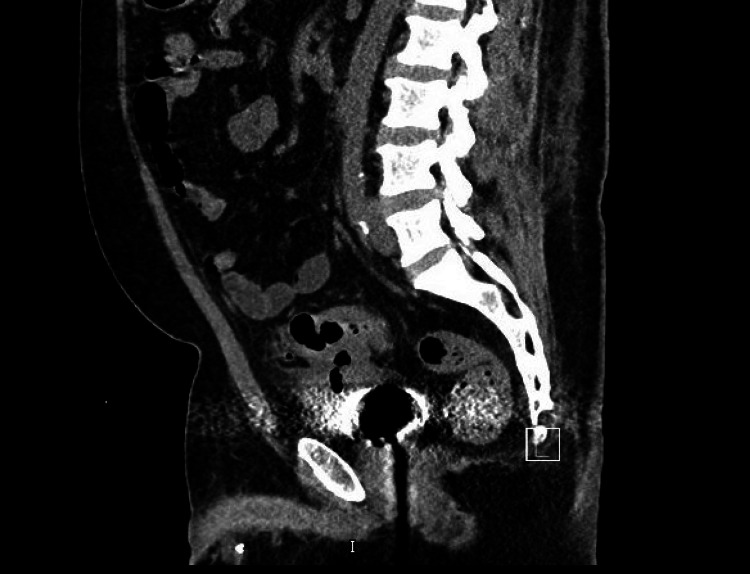
Repeat CT of the abdomen and pelvis Gas is seen in the urinary bladder, raising suspicion for a CVF CVF: colovesical fistula

**Figure 7:**
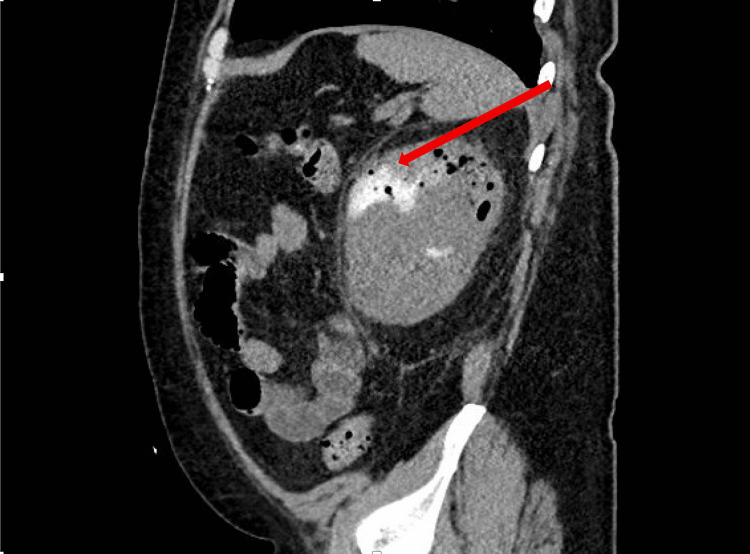
CT scan showing emphysematous pyelonephritis developing into a renal abscess (red arrow)

The patient was initially treated with ceftriaxone on admission, but antibiotics were broadened to ciprofloxacin, metronidazole, and meropenem with these findings. He subsequently developed consecutive days of fever up to 100.4 °F, which prompted a second computed tomography three days later. Figure 6 shows the presence of a possible CVF with increasing volume of gas within the urinary bladder. Figure 7 shows the previous findings of emphysematous pyelonephritis, as well as concern for developing a renal abscess.

**Figure 8:**
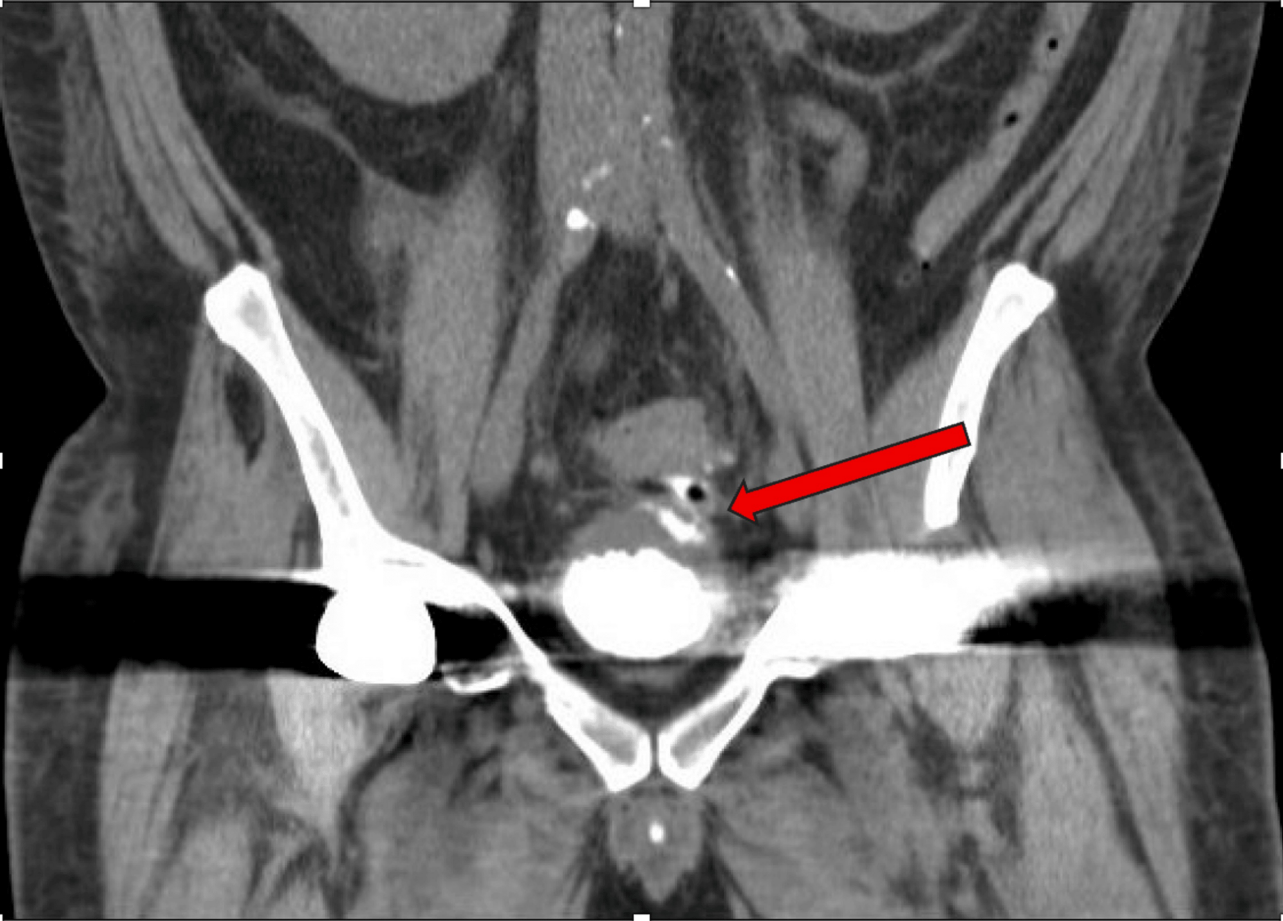
CT pelvis cystogram with contrast Contrast is seen traveling from the bladder to the colon (red arrow)

A CVF was confirmed on Figure 8 with a computed tomography cystogram of the pelvis.

The patient underwent successful nephrostomy tube placement for source control and CVF repair with general surgery and urology. Unfortunately, the patient experienced recurring fevers for 31 consecutive days, attributed to the left renal abscess that contributed to the patient’s kidney disease. Notably, the patient was initiated on hemodialysis but started to produce a robust amount of urine, averaging 1.5 liters over 24 hours within five days of his hospital stay, and dialysis was ultimately stopped. Biopsy results were notable for diffuse severe tubulointerstitial inflammation with tubular microabscesses consistent with pyelonephritis and marked fibrosis and arteriosclerosis. Discharge was deemed appropriate after two consecutive days of normal body temperature. He was discharged on oral Levaquin for 28 days with nephrology and urology follow-up. Serum creatinine at time of discharge was 5 mg/dl. Unfortunately, kidney function has declined significantly. His most recent creatinine is 3.06 mg/dL, with a glomerular filtration rate of 24 mL/min/1.73 m². His baseline creatinine prior to hospitalization was 1.2 mg/dL. Patient underwent an outpatient renal scan with urology, which demonstrated overall poor functioning of the left kidney. The left kidney was estimated to contribute approximately 20% of overall renal function. The urologist also noted increased risk of future complications, including recurrent infections, for which the patient may ultimately require nephrectomy in the future. 

## Discussion

CVF is defined as an abnormal connection between the colon and the bladder. This patient case is extremely rare, given the extension of infection without symptoms of CVF. There have been no other cases reported of CVF resulting in EPN and acute renal failure. Around 66% of CVFs are caused by complicated diverticulitis [5]. There was no history of diverticulosis in this patient, nor did they present with abdominal pain, pneumaturia, or fecaluria, which is what makes this case so unique. Furthermore, they didn’t have any other risk factors that could cause CVF formation, such as inflammatory bowel disease (particularly Crohn’s disease), gynecologic or colorectal malignancy, or iatrogenic injury. 

There have been multiple theories regarding the pathophysiology behind fistula formation due to diverticulitis. One of the more common ones involves fecaliths obstructing the lumen of the colon and infecting diverticula. However, other theories exist; Seeras et al. suggest that abnormal peristalsis increases intraluminal pressure, which directs force radially into the diverticula, causing micro- and macro-perforations that can lead to diverticular abscesses [5]. Phlegmon and abscesses can rupture into adjacent organs, leading to inflammation and abnormal epithelialization [6,7]. Zullo describes the ischemic theory, which involves compression of the vasa recta in the neck of the diverticula due to strong contractions in the colon [8]. In the case of malignancy, Crohn's disease, or iatrogenic causes, the pathophysiology differs. It is not uncommon for the diagnosis to be delayed after symptoms have begun [9]. In this patient, diverticulitis was the ultimate cause of the formation of the CVF and presentation to the emergency department. Diverticulosis may occur throughout the colon, but the sigmoid colon is the most common area [10]. Bacteriuria is common in patients with CVF secondary to sigmoid diverticulitis and was found in 100% of patients in a retrospective study that followed 10 cases of CVF [3]. The most common symptoms include pneumaturia and fecaluria, which are present in 70-90% and 50-70% of cases, respectively [1-4]. Other symptoms include dysuria, polyuria, urgency, hematuria, and suprapubic pain [2]. Although bacteria were present in the patient’s urinalysis, he did not initially present with infectious symptoms, nor did he have a history of diverticulosis. Instead, he presented with symptoms of acute renal failure. 

Computed tomography and cystoscopy are the most common imaging modalities used in determining the presence of CVF. In this patient, the initial CT scan showed diverticulitis and emphysematous pyelonephritis; the latter being the result of bacterial spread from the colon to the bladder and up to the ureters and kidneys. This case was complex because the patient did not exhibit common CVF symptoms, and initial imaging did not reveal a fistula.

There are other imaging modalities that reveal the presence of a CVF. A systematic review by Zizzo et al., covering 1982 to 2019, found that abdominal CT, colonoscopy, and cystoscopy were the most commonly used diagnostic modalities [11]. Another study by Holroyd et al. from 2003-2009 consisted of 37 patients who underwent an average of 2.78 imaging modalities in order to capture a CVF signifying the importance of multiple imaging studies. The most frequent modality performed was a CT scan, which had a sensitivity of 76.5% in diagnosing CVF [12]. This same study showed that colonoscopy or flexible sigmoidoscopy had a sensitivity of 64.7% when detecting CVF, but similar accuracy compared to CT. Cystoscopy was performed in less than half of the patients, with an overall lower sensitivity of 40% when detecting a vesicular fistulous orifice [12]. In this patient, a fistula wasn’t visualized until their second CT scan three days later due to the presence of an unremitting fever. A subsequent cystography with contrast was performed, which confirmed CVF. 

There are different treatment options for CVF depending on the extension of infection and presenting symptoms. Surgical treatments include laparoscopic vs open bowel resection with primary anastomosis, double-barreled colostomy, and sigmoidectomy with ileostomy. For diverticular disease, bowel resection with primary anastomosis is the most common surgical treatment [3]. This patient underwent open bowel resection with primary anastomosis and bladder repair. Another common comparison is the open versus laparoscopic approach to surgical treatment. Laparoscopy is less invasive and reduces trauma to the skin, but a systematic review by Trejo-Avila et al. with 227 patients from 2014-2020 showed that a shorter hospital stay was the only difference between the laparoscopic and open approach. There was no difference in anastomotic leaks, infections, stoma rates, or mortality when compared with open surgery [13].

## Conclusions

This case highlights a previously unknown and severe presentation of colovesical fistula manifesting as emphysematous pyelonephritis and acute renal failure in a middle-aged man. It underscores the importance of maintaining a high index of suspicion for fistula formation in patients with persistent urinary tract infections, renal dysfunction, and colonic pathology, even in the absence of classic symptoms like pneumaturia. It also highlights CVF as an unexpected cause of proteinuria. Prompt recognition and multidisciplinary management are essential to prevent long-term morbidity. 
